# Striatal temporal interference stimulation alleviates motor deficits and confers neuroprotection in an MPTP-induced mouse model of Parkinson’s disease with associated improvements in mitochondrial homeostasis

**DOI:** 10.1016/j.neurot.2026.e01064

**Published:** 2026-09-08

**Authors:** Jin Peng, Ziyu Wang, Yu Liu, Xiaohui Wang

**Affiliations:** a School of Exercise and Health, Shanghai University of Sport, Shanghai, 200438, China; b Key Laboratory of Exercise and Health Sciences of Ministry of Education, Shanghai University of Sport, Shanghai, China

**Keywords:** Temporal interference stimulation (TI), Parkinson’s disease (PD), Neuroprotection, Mitochondrial homeostasis, α-synuclein

## Abstract

Temporal interference (TI) stimulation is an emerging noninvasive neuromodulation approach capable of selectively targeting deep brain structures, but its therapeutic effects and underlying mechanisms in Parkinson’s disease (PD) remain unclear. Here, we evaluated striatal TI stimulation in an MPTP-induced mouse model of PD. TI stimulation using 2 and 2.02 kHz carrier frequencies, a 20 Hz offset, and a current of 150 μA was delivered for 10 min/day, with 30 s ramp-up and ramp-down, for 7 or 14 consecutive days. We found that TI stimulation significantly reduced motor deficits, with greater behavioral benefit observed after 14 days of stimulation, and attenuated dopaminergic neurodegeneration and α-synuclein accumulation. Meanwhile, TI stimulation improved multiple aspects of mitochondrial homeostasis, including mitochondrial morphology and dynamics, PINK1/Parkin-mediated mitophagy, complex I activity, and mitochondria-dependent apoptotic signaling. Importantly, pharmacological inhibition with Mdivi-1 largely attenuated the behavioral, neuropathological, and mitochondrial effects of TI stimulation, suggesting that mitochondrial quality-control pathways may contribute to TI-associated neuroprotection. Together, these findings indicate that striatal TI stimulation ameliorates motor impairment and exerts neuroprotective effects in parkinsonian mice, with associated improvements in mitochondrial homeostasis, and support TI stimulation as a promising noninvasive therapeutic strategy for PD.

## Introduction

Parkinson’s disease (PD) is the second most common age-related neurodegenerative disorder after Alzheimer’s disease and is projected to affect nearly 10 million people worldwide by 2030 [1]. It is clinically characterized by progressive motor dysfunction, including bradykinesia, resting tremor, and rigidity, together with diverse non-motor symptoms [2]. Pathologically, PD is marked by progressive degeneration of dopaminergic neurons in the substantia nigra pars compacta (SNpc) and abnormal accumulation of α-synuclein (α-syn) [3]. Although levodopa remains the cornerstone of symptomatic treatment, its long-term efficacy is compromised by disabling motor complications, particularly levodopa-induced dyskinesia [4]. Deep brain stimulation (DBS) provides an effective alternative for selected patients [5], but its invasiveness and procedure-related risks restrict broader clinical application [6]. Therefore, developing safe and effective noninvasive neuromodulation strategies for PD remains an important unmet need.

Temporal interference (TI) stimulation is a recently developed noninvasive neuromodulation approach that enables selective modulation of deep brain structures by delivering two sets of high-frequency (*f* > 1 kHz) sinusoidal currents with a small frequency difference, thereby generating a low-frequency envelope at the target region [7]. Previous studies in healthy humans and rodents have shown that TI stimulation can enhance motor performance and motor learning when targeting motor-related brain regions, including the primary motor cortex (M1) and striatum [[8], [9], [10], [11]], suggesting that TI stimulation may have therapeutic potential in disorders involving dysfunctional motor circuits.

In recent years, TI stimulation has emerged as a feasible intervention for PD-related motor dysfunction [[12], [13], [14]]. Early clinical studies have suggested that TI stimulation targeting deep basal ganglia structures, such as the globus pallidus internus (GPi) and subthalamic nucleus (STN), was well tolerated and may improve motor symptoms and gait control in patients with PD [[12], [13], [14]]. However, despite these encouraging findings, the biological mechanisms underlying TI-induced benefits in PD remain largely unclear. In particular, it is not known whether TI stimulation exerts neuroprotective effects beyond symptomatic motor improvement, nor whether such effects are linked to restoration of key pathogenic processes in PD.

Mitochondrial dysfunction is a central contributor to PD pathogenesis in both sporadic and genetic forms of the disease [15,16]. Defects in mitochondrial dynamics, mitophagy, and respiratory chain function lead to impaired adenosine triphosphate (ATP) production, excessive reactive oxygen species generation, and increased neuronal vulnerability [17,18]. Dopaminergic neurons are particularly susceptible to mitochondrial oxidant stress because of their high energy demand and complex axonal architecture [19]. In addition, mitochondrial dysfunction is closely linked to α-syn pathology and apoptosis, forming a pathogenic network that promotes neurodegeneration [3,18]. Therefore, restoration of mitochondrial homeostasis represents a biologically meaningful and potentially disease-modifying therapeutic strategy in PD.

In the present study, we investigated whether striatal TI stimulation alleviates motor deficits and dopaminergic neurodegeneration in a PD mouse model induced by 1-methyl-4-phenyl-1,2,3,6-tetrahydropyridine (MPTP), and whether mitochondrial homeostasis-related processes contribute to these effects. To address this, mice received striatal TI stimulation for 7 or 14 consecutive days after MPTP administration. The stimulation parameters used here (2 and 2.02 kHz carrier frequencies, Δ*f* = 20 Hz, 150 μA current, 30 s ramp-up/down, 10 min/day) were selected based on prior TI studies in humans [9,[20], [21], [22], [23]] and our previous mouse work showing motor-related neuromodulatory effects [10,11,24]. We then assessed motor behavior, dopaminergic neuronal survival, α-syn accumulation, mitochondrial morphology and dynamics, mitophagy, complex I activity, and mitochondria-dependent apoptosis. In addition, Mdivi-1 was used to probe the involvement of mitochondrial quality-control-related mechanisms in TI-mediated neuroprotection.

## Materials and Methods

### Animals and ethics

Male C57BL/6 J mice (7–8 weeks old; 22–25 g; total n = 258) were purchased from GemPharmatech Co., Ltd (Nanjing, China). All experimental procedures were approved by the Ethics Committee of Shanghai University of Sport (Approval No. 102772025DW019) and were conducted in accordance with the ARRIVE guidelines [25]. Mice were housed under a 12-h light/12-h dark cycle with ad libitum access to food and water. The temperature was maintained at 21 ± 2 °C with a humidity of 40–60%.

### Experimental design and grouping

To evaluate the effects of striatal TI stimulation on MPTP-induced motor deficits, neuropathological changes, and mitochondrial abnormalities, mice were randomly assigned to four groups: (1) Con + sham, receiving surgical procedures and sham stimulation; (2) Con + TI, receiving TI stimulation; (3) MPTP + sham, receiving MPTP injection, surgical procedures, and sham stimulation; and (4) MPTP + TI, receiving MPTP injection and TI stimulation. Sham stimulation was defined as identical anesthesia, handling, electrode placement and connection, and session duration, but with no current delivered.

To investigate the mechanisms underlying the beneficial effects of TI stimulation on motor impairments, mice were randomly assigned to five groups: (1) Con + sham; (2) MPTP + sham; (3) MPTP + TI; (4) MPTP + TI + Mdivi-1, receiving MPTP injection, Mdivi-1 treatment, and TI stimulation; and (5) MPTP + Mdivi-1, receiving MPTP injection, Mdivi-1 treatment, surgical procedures, and sham stimulation.

### MPTP administration and Mdivi-1 treatment

**Fig. 1:**
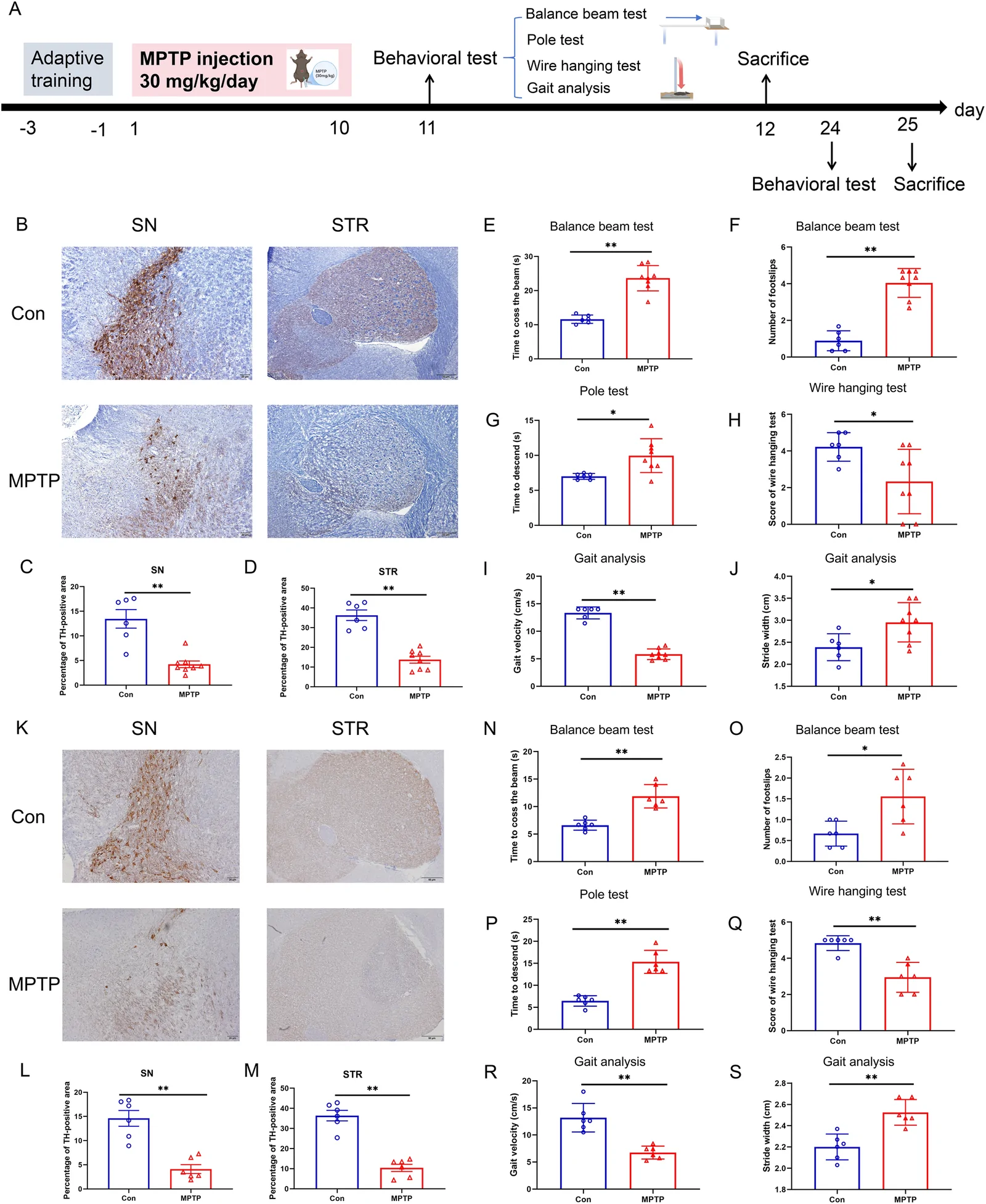
**Establishment of an MPTP-induced mouse model of PD.** (A) Schematic of experimental timeline. (B–D) IHC and quantification of TH-positive neurons in the SN and striatum (STR) 1 day after the final MPTP injection. N = 6–8. (E–J) Behavioral tests performed 1 day after the final MPTP injection. N = 6–8. (K–M) IHC and quantification of TH-positive neurons in the SN and STR 14 days after the final MPTP injection. N = 6. (N–S) Behavioral tests performed 14 days after the final MPTP injection. N = 6. All data are presented as mean ± SD. ∗P < 0.05, ∗∗P < 0.01, vs Con group.

To establish the PD mouse model, mice received daily intraperitoneal injections of MPTP (30 mg/kg; MedChemExpress, USA) for 10 consecutive days, while control mice received an equivalent volume of saline [26,27] (Fig. 1A). For mechanistic experiments, mice received daily intraperitoneal injections of Mdivi-1 (30 mg/kg; MedChemExpress, USA) for 14 consecutive days. Mdivi-1 was used as a pharmacological tool to interfere with mitochondrial quality-control-related processes [[28], [29], [30]].

### Electrode fixation and TI stimulation protocol

**Fig. 2:**
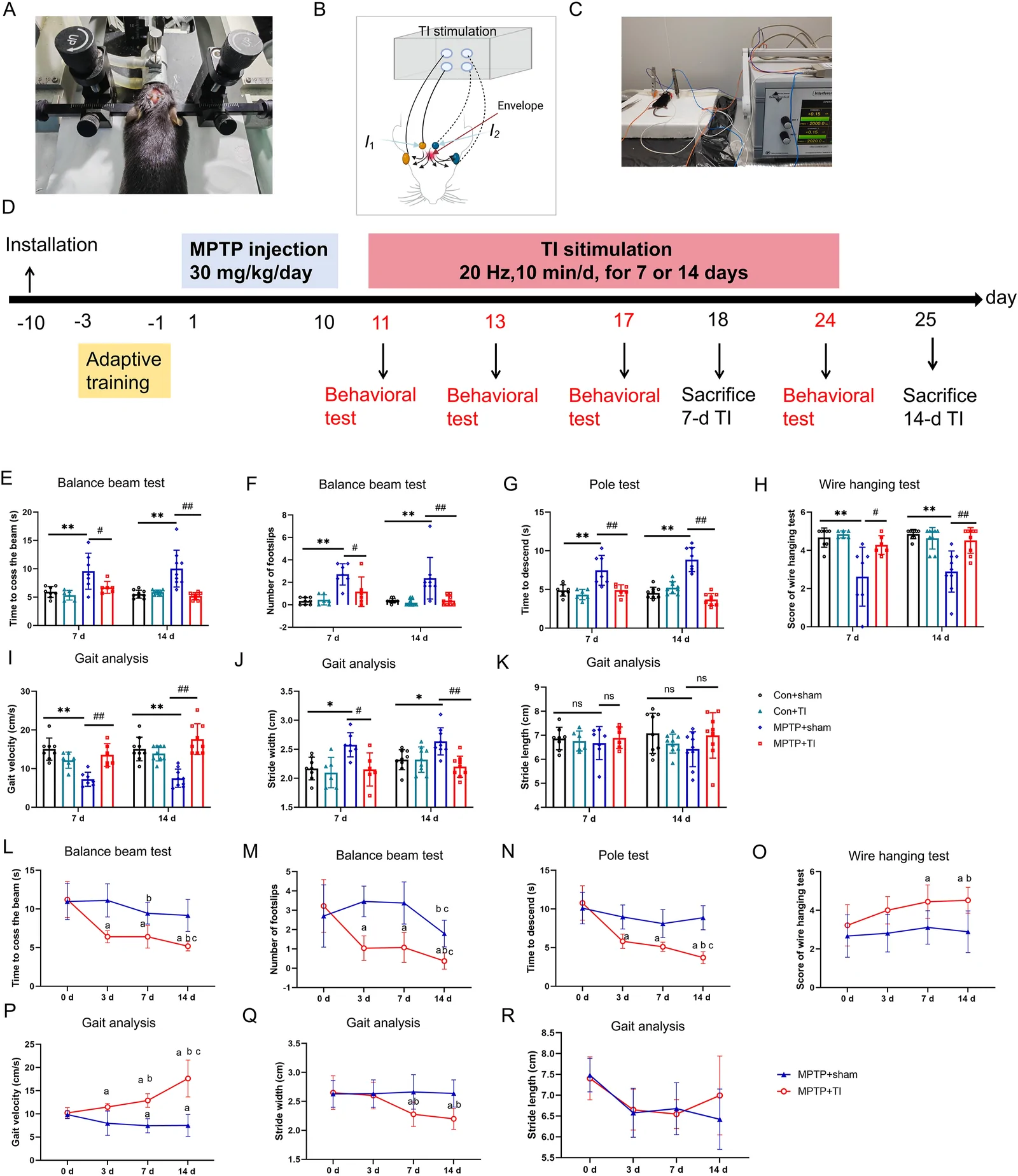
**Striatal TI stimulation ameliorates motor deficits in MPTP-treated mice.** (A) Skull electrode coordinate positioning. (B) Schematic of TI stimulation. Currents *I*_1_ and *I*_2_ are simultaneously applied to the brain via electrodes at kilohertz frequencies *f*_1_ and *f*_2_ (*f*_1_+Δ*f*). The superposition of the two kHz currents is intended to generate an amplitude-modulated interference field with an envelope frequency of Δ*f* in the deep target region. (C) Photography of a mouse receiving TI stimulation. (D) Schematic of experimental timeline. (E–K) Behavioral tests performed on day 7 and day 14 of TI stimulation. N = 6–9. (L–R) Behavioral tests performed on days 0, 3, 7, and 14 of TI stimulation. N = 6–9. All data are presented as mean ± SD. ∗P < 0.05, ∗∗P < 0.01, vs Con + sham group; #p < 0.05, ##p < 0.01, vs MPTP + sham group. A p < 0.05, vs day 0; b p < 0.05, vs day 3; c p < 0.05, vs day 7.

Mice were anesthetized with isoflurane (1%–2%) and fixed on the stereotaxic apparatus. Then, remove the scalp and underlying tissues, and mark the position of Bregma. Skull electrodes (internal diameter 1.0 mm) were fixed onto the mouse skull and secured by dental cement. Skull electrodes should not penetrate the skull bone. Two high-frequency electrically isolated currents, *I*_1_ and *I*_2_, are simultaneously applied to the brain via electrodes. The coordinates of skull electrodes (relative to bregma) are as follows: (1) *I*_1_ electrode: AP -1.5 mm, ML +2.75 mm; (2) *I*_2_ electrode: AP -1.5 mm, ML -0.25 mm [7,11] (Fig. 2A). *I*_1_ and *I*_2_ skull electrodes were paired with electrodes (diameter 10 mm) placed on the ipsilateral cheek. After the operation, the mice were allowed a week-long recovery period.

TI stimulation was applied to anesthetized mice (with 1%–2% isoflurane) utilizing a battery-powered current stimulator (Interferential Neuromodulation System, Soterix Medical, USA) [24] (Fig. 2C). Striatal TI stimulation was performed 10 min per day for 7 or 14 consecutive days. The stimulation parameters are as follows: (1) *I*_1_: 2 kHz, 150 μA; (2) *I*_2_: 2.02 kHz, 150 μA; (3) ramp duration: 30 s ramp-up and 30 s ramp-down; (4) waveform: Β-SINE [11,24]. Sham stimulation consisted of the same anesthesia, handling, electrode placement and connection, and session duration as active TI stimulation, but with the stimulator output set to 0 μA throughout the session. Therefore, sham mice received neither kilohertz carrier stimulation nor a low-frequency interference envelope.

### Behavioral tests

All behavioral tests were conducted by the same two trained investigators, who were aware of the group assignments. Standardized testing procedures were applied throughout the study, and behavioral assessments were performed at approximately the same time of day across experimental sessions to minimize procedural and temporal variability.

#### Balance beam test

The balance beam test was used to assess motor coordination and balance [31]. The beam (100 cm long, 0.6 cm in diameter) was elevated 50 cm above the ground. Mice were placed at one end and allowed to traverse the beam to a closed box. The time to traverse the beam and the number of footslips were recorded. Each mouse performed three trials at 1-min intervals, and the mean value was used for analysis.

#### Pole test

The pole test was used to evaluate bradykinesia [31]. Mice were placed head-up at the top of a rough-surfaced pole (50 cm long, 1 cm in diameter), and the time spent climbing down to the bottom of the pole was recorded. Each mouse performed three trials at 1-min intervals, and the mean value was used for analysis.

#### Wire hanging test

The wire hanging test was used to evaluate muscle endurance and rigidity [32]. Mice were suspended by their forepaws from the center of a metal wire (50 cm long, 0.2 cm in diameter, 20 cm above the ground). Performance was scored as follows: 0, fall off; 1, hung by both forepaws; 2, hung by both forepaws and attempted to climb; 3, hung by both forepaws and one or both hindpaws; 4, hung by all four paws and tail; 5, escaped. The maximum test duration was 30 s per mouse.

#### Gait analysis

Gait analysis was used to evaluate bradykinesia and ataxia [33]. The white paper (42 cm long, 15 cm wide) and ink were prepared in advance. Mice were trained to walk through the white paper passage, leaving their footprints. The time taken to walk through the passage was recorded. The footprints were analyzed. Gait velocity, stride width, and stride length were used for analysis.

### Immunohistochemistry (IHC)

Brain tissues were collected by transcardial perfusion on the day following completion of the experimental protocol. Coronal sections (20 μm thickness) underwent permeabilization with 0.3% Triton X-100, followed by incubation in endogenous peroxidase blocking buffer and blocking with 10% goat serum. Sections were then incubated overnight at 4 °C with rabbit anti-tyrosine hydroxylase (TH) antibody (1:150; Cell Signaling Technology, USA), followed by goat anti-rabbit HRP IgG (1:50; Beyotime Biotechnology, China) for 1 h at 37 °C. Sections were subsequently incubated in DAB, hematoxylin, and 1% hydrochloric acid-alcohol, dehydrated through graded ethanol, cleared in xylene, and sealed with Rhamsan gum. Images were captured with an Olympus or a Nikon microscope (Japan). The percentage of TH-positive area in the substantia nigra (SN) and striatum was quantified using ImageJ software. For each mouse, three coronal brain sections containing the corresponding region of interest were analyzed. The percentage of TH-positive area was measured separately in each section, and the values obtained from the three sections from the same mouse were averaged to generate a single representative value for each brain region in that animal. 6–8 mice were included in each group. Each animal was considered an independent biological replicate and the statistical unit, and statistical analyses were performed using the animal-level mean values.

### Immunofluorescent (IF)

#### Tissue collection

For c-Fos analysis, mice received a single session of TI stimulation and were subsequently maintained in a dark environment for 90 min, during which external environmental stimulation was minimized. The mice were then transcardially perfused for brain tissue collection.

For dynamin-related protein 1 (Drp1) and optic atrophy 1 (OPA1) analyses, brain tissues were collected by transcardial perfusion on the day following completion of the experimental protocol. Following perfusion, brain tissues for all three markers were processed using the same procedures for subsequent IF staining.

#### If staining

Coronal sections (20 μm thickness) were permeabilized using 0.3% Triton X-100, followed by blocking with 10% goat serum. Sections were then incubated overnight at 4 °C with primary antibodies, including c-Fos (1:150; Cell Signaling Technology, USA), Drp1 (1:100; Cell Signaling Technology, USA), and OPA1 (1:100; Proteintech, China). The next day, sections were incubated with Alexa Fluor™ 594-conjugated secondary antibody (1:500; Thermo Fisher Scientific, USA) for 2 h at room temperature. Nuclear staining was performed with DAPI, and sections were mounted using antifade medium. Fluorescence images were acquired using a confocal microscope (Zeiss, Germany).

#### Image acquisition and quantitative analysis

For c-Fos analysis, fluorescence intensity was quantified in both the striatum and cortex using ImageJ software, whereas Drp1 and OPA1 fluorescence intensities were quantified in the striatum. For each mouse, three coronal brain sections containing the corresponding regions of interest were analyzed using ImageJ. Fluorescence intensity was measured separately in each section, and the values obtained from the three sections from the same mouse were averaged to generate a single representative value for each brain region in that animal. Each animal was considered an independent biological replicate, with n = 3 mice per group for c-Fos analysis and n = 6 mice per group for Drp1 and OPA1 analyses. Statistical analyses were performed using these animal-level mean fluorescence intensity values. ImageJ analysis was performed without blinding to experimental group allocation.

### Western blot (WB)

Striatal tissues were homogenized in RIPA buffer containing protease and phosphatase inhibitors. Protein concentrations were measured using a BCA assay kit. Equal amounts of protein were separated on 12.5% SDS-PAGE gels and transferred onto PVDF membranes. Membranes were blocked in 5% non-fat milk and incubated overnight at 4 °C with primary antibodies, including α-syn (1:1000), Drp1 (1:1000), PTEN-induced kinase 1 (PINK1; 1:2000), Parkin (1:2000), sequestosome 1 (P62/SQSTM1; 1:1000), microtubule-associated protein 1 light chain 3 II (LC3 II; 1:3000), Beclin 1 (1:1000), BCL-2-associated X protein (Bax; 1:1000), and Caspase-3 (Cas-3; 1:1000), all from Cell Signaling Technology (USA); as well as B-cell lymphoma 2 (Bcl-2; 1:1000), OPA1 (1:1000), and glyceraldehyde-3-phosphate dehydrogenase (Gapdh; 1:2000), all from Proteintech (China). After that, membranes were incubated with HRP-conjugated secondary antibodies for 1 h at room temperature. Protein bands were visualized using an automatic chemiluminescence apparatus (Tanon Biotechnology, China) and signal intensity was measured using ImageJ software.

### Transmission electron microscopy (TEM)

The striatum was isolated and fixed with the electron microscopy fixative. The striatum was dehydrated, infiltrated, embedded, polymerized, ultrathin-sectioned (70 nm), and then stained. Images were captured by a transmission electron microscopy (Hitachi, Japan).

### Assessment of complex I activity and ATP levels

Mitochondrial complex I activity and ATP levels in the striatum were quantified using commercial assay kits (Solarbio, China) following the manufacturer’s instructions. Briefly, mitochondrial homogenates were added to the corresponding reaction buffers, and absorbance was measured at 340 nm using a prewarmed microplate reader (CLARIOstar, Germany). Complex I activity was expressed as nmol/min/mg protein. ATP levels were measured in striatal supernatants. Supernatants were mixed with the provided reaction buffer, and ATP standards were prepared in parallel. Absorbance at 340 nm was measured using the same prewarmed microplate reader.

### Proteomics and analysis

Striatal tissues were subjected to proteomics by the APExBIO Technology LLC (Shanghai, China) and data analysis. The overall process of the proteome is as follows: (1) Proteins in the samples underwent protein extraction, enzymatic digestion, and peptide desalting, and thereby a peptide detection solution suitable for mass spectrometry analysis was made. (2) The peptides were detected and analyzed by liquid chromatography-tandem mass spectrometry (LC-MS/MS).

### Safety evaluation

Hematoxylin-eosin (HE) and Nissl staining were performed to evaluate the safety of TI stimulation [24]. Images were captured by an Olympus microscope (Japan).

### Statistical analysis

All data are expressed as mean ± standard deviation (SD). Data analysis and visualization were performed with SPSS 26.0 and GraphPad Prism 8. Statistical analysis was performed via unpaired *t*-test, two-way ANOVA plus Tukey’s post hoc test, and one-way repeated-measures ANOVA for parametric analysis; and Mann-Whitney test, Kruskal-Wallis test, and Friedman test for non-parametric test. Significant difference was assessed as p < 0.05.

## Results

### Establishment of an MPTP-induced mouse model of PD

A subacute PD mouse model was established by repeated MPTP administration, which is known to lesion the nigrostriatal dopaminergic pathway and recapitulate key pathological features of PD [26]. We first assessed dopaminergic neurodegeneration and motor function 1 day after the final MPTP injection (Fig. 1A). MPTP-treated mice showed a marked reduction in TH-positive neurons in the SN and TH-positive neurites in the striatum compared with control mice (Fig. 1B–D). Consistent with this neuropathology, MPTP-treated mice exhibited significant motor deficits in the balance beam test, pole test, wire hanging test and gait analysis, as reflected by increased beam-crossing time and footslips, prolonged pole-descending time, reduced wire hanging performance, decreased gait velocity and increased stride width (Fig. 1E–J). Notably, these abnormalities remained evident 14 days after the final injection, with MPTP-treated mice continuing to exhibit both motor impairment and nigrostriatal dopaminergic loss (Fig. 1K–S). Together, these findings support the successful establishment of a stable PD-like mouse model for subsequent TI stimulation experiments.

### Striatal TI stimulation ameliorates motor deficits in MPTP-treated mice

To determine whether striatal TI stimulation alleviates motor deficits in MPTP-treated mice, we performed behavioral assessments during the stimulation period (Fig. 2D). Both stimulation regimens significantly improved motor performance, as reflected by reduced beam-crossing time and footslips, shortened pole-descending time, improved wire hanging performance, increased gait velocity and reduced stride width relative to MPTP-treated mice without stimulation (Fig. 2E–K). Notably, these effects became more pronounced with prolonged stimulation, with 14 days of TI stimulation consistently producing greater behavioral benefit than 7 days of stimulation (Fig. 2L-R). These data show that striatal TI stimulation ameliorates MPTP-induced motor deficits in a duration-dependent manner.

### Striatal TI stimulation attenuates nigrostriatal dopaminergic degeneration and reduces α-syn accumulation in MPTP-treated mice

**Fig. 3:**
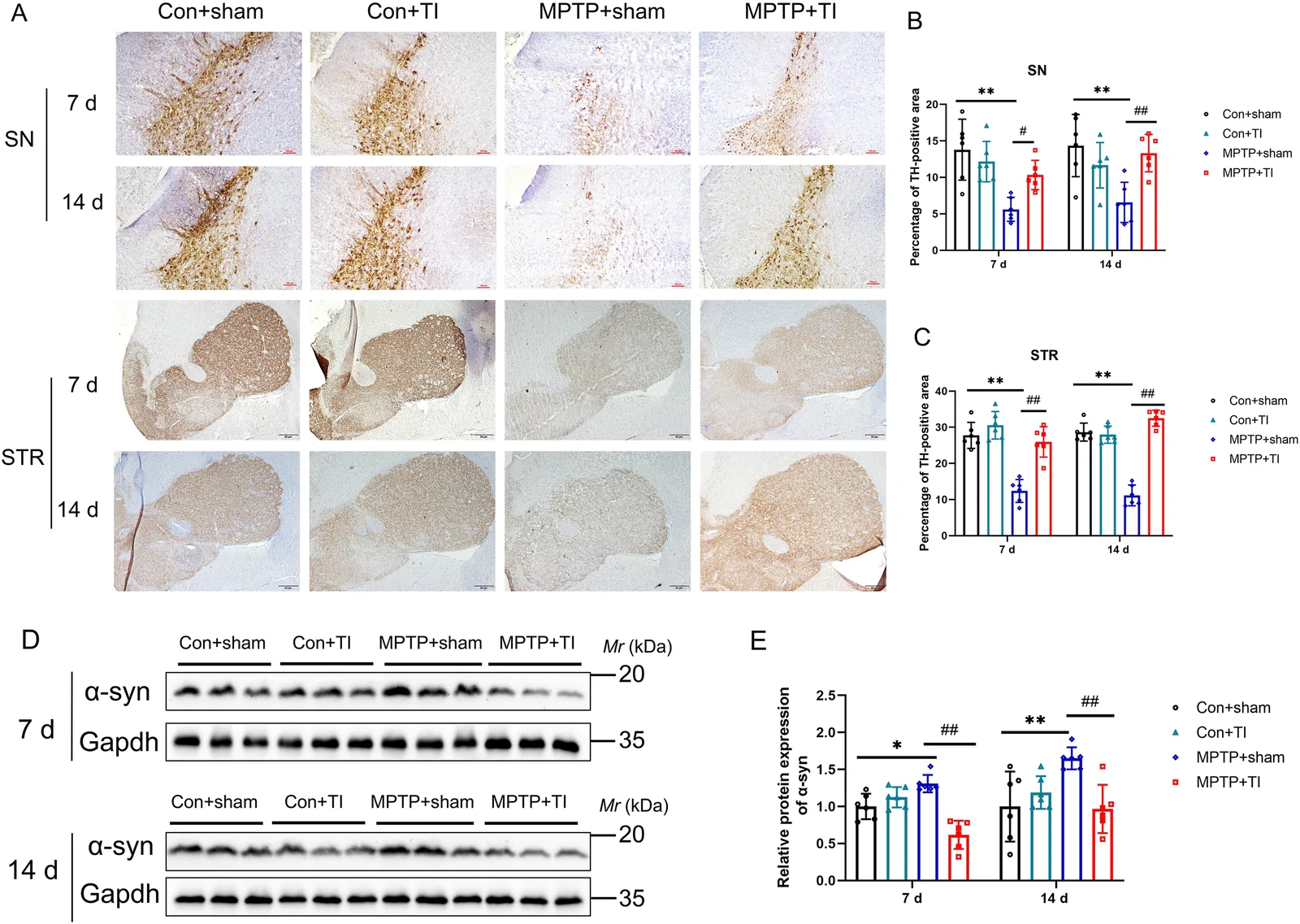
**Striatal TI stimulation attenuates nigrostriatal dopaminergic degeneration and reduces α-syn accumulation in MPTP-treated mice.** (A–C) IHC and quantification of TH-positive neurons in the SN and striatum. N = 6. (D, E) WB and quantification of striatal α-syn expression. N = 6. All data are presented as mean ± SD. ∗P < 0.05, ∗∗P < 0.01, vs Con + sham group; #p < 0.05, ##p < 0.01, vs MPTP + sham group.

To determine whether the motor benefits of TI stimulation were accompanied by neuropathological improvement, we assessed nigrostriatal dopaminergic degeneration and α-syn expression in MPTP-treated mice. Both 7-day and 14-day striatal TI stimulation attenuated the loss of TH-positive neurons in the SN and TH-positive neurites in the striatum (Fig. 3A–C). Notably, 14 days of TI stimulation produced greater preservation of TH-positive neurons than 7 days of stimulation in both the SN (102.70% versus 83.79%) and striatum (191.43% versus 109.63%) of MPTP-treated mice. In parallel, α-syn expression was markedly elevated in the striatum of MPTP-treated mice, but this increase was significantly reduced by both TI stimulation paradigms (Fig. 3D and E). These results show that striatal TI stimulation mitigates dopaminergic degeneration and α-syn accumulation in MPTP-treated mice.

### Striatal TI stimulation regulates mitochondrial homeostasis-related pathways in normal mice

**Fig. 4:**
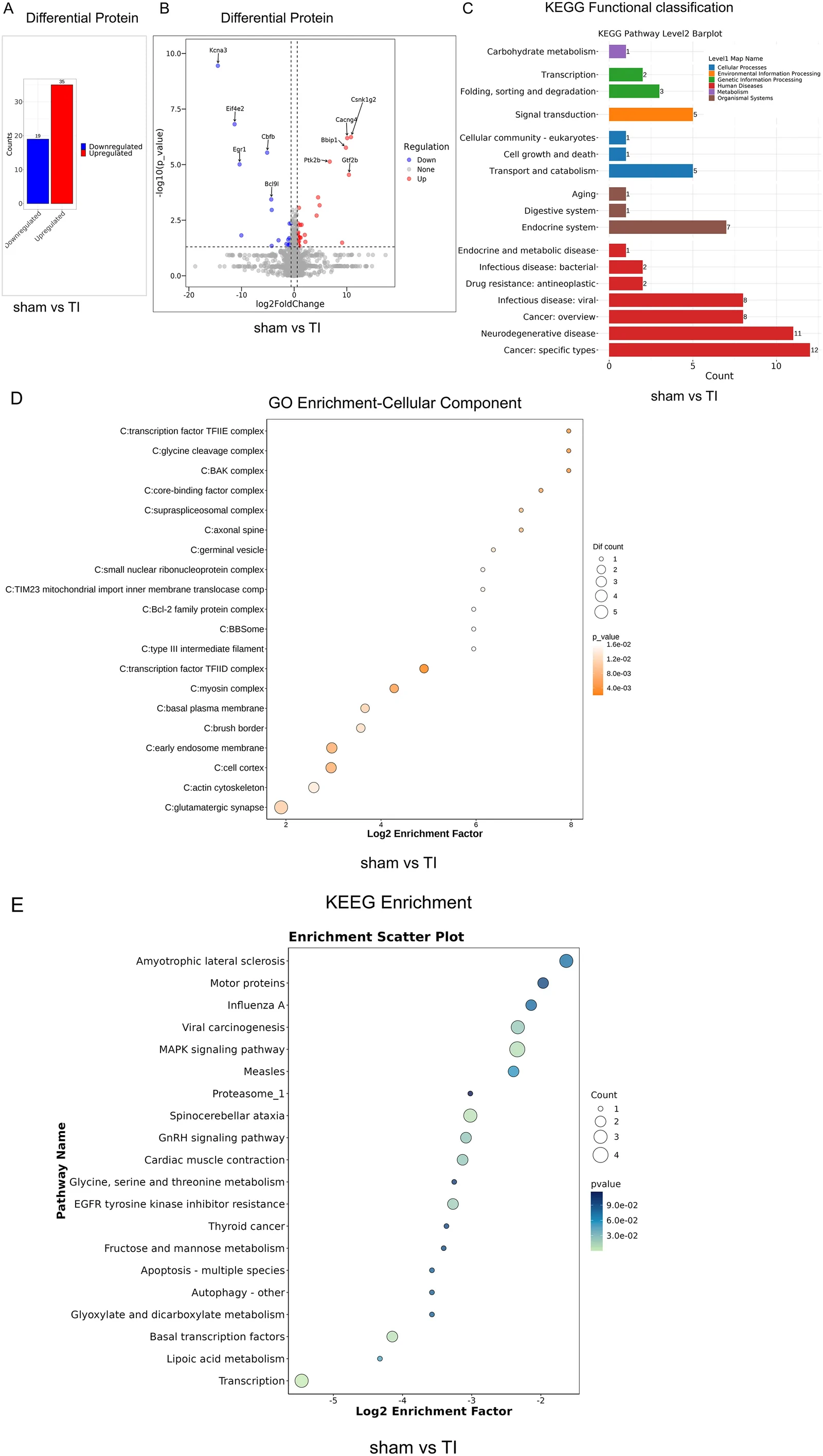
**Striatal TI stimulation regulates mitochondrial homeostasis-related pathways in normal mice.** Barplot (A) and volcano plot (B) of differentially expressed proteins in the striatum of mice after 7-d TI stimulation compared to the sham group. Significantly upregulated proteins are shown in red (P < 0.05 and fold change >1.5). Significantly downregulated proteins are labeled blue (P < 0.05 and fold change <1/1.5). (C) KEGG functional classification analysis of differentially expressed proteins. (D) GO enrichment analysis of differentially expressed proteins. (E) KEGG enrichment analysis of differentially expressed proteins. N = 3.

To investigate molecular changes induced by TI stimulation in normal mice, we performed proteomic analysis of striatal tissue from the Con + sham and Con + TI groups. TI stimulation significantly altered 54 proteins (Fig. 4A and B), of which 11 were classified as neurodegenerative disease-related by KEGG analysis (Fig. 4C). Notably, several upregulated proteins were linked to mitochondrial structure and quality control, including reactive oxygen species modulator 1 (Romo 1), an important redox-dependent regulator of mitochondrial dynamics [34], and autophagy-related protein 2 homolog A (Atg2a), which participates in mitophagy (Supplementary Data 1). By contrast, Bcl-2 homologous antagonist/killer 1 (Bak 1), a pro-apoptotic protein localized to the mitochondrial outer membrane [35], was downregulated after TI stimulation (Supplementary Data 1).

GO enrichment analysis revealed significant enrichment of mitochondrial- and apoptosis-related cellular components, including the TIM23 mitochondrial import inner membrane translocase complex, BAK complex and Bcl-2 family protein complex (Fig. 4D). KEGG enrichment analysis further identified pathways related to motor proteins, autophagy and apoptosis (Fig. 4E). Given the established roles of these processes in mitochondrial transport, dynamics and turnover [36], these findings suggest that TI stimulation may engage mitochondrial homeostasis-related pathways in the striatum under physiological conditions, supporting further investigation in the PD model.

### Striatal TI stimulation normalizes mitochondrial morphology and dynamics in MPTP-treated mice

**Fig. 5:**
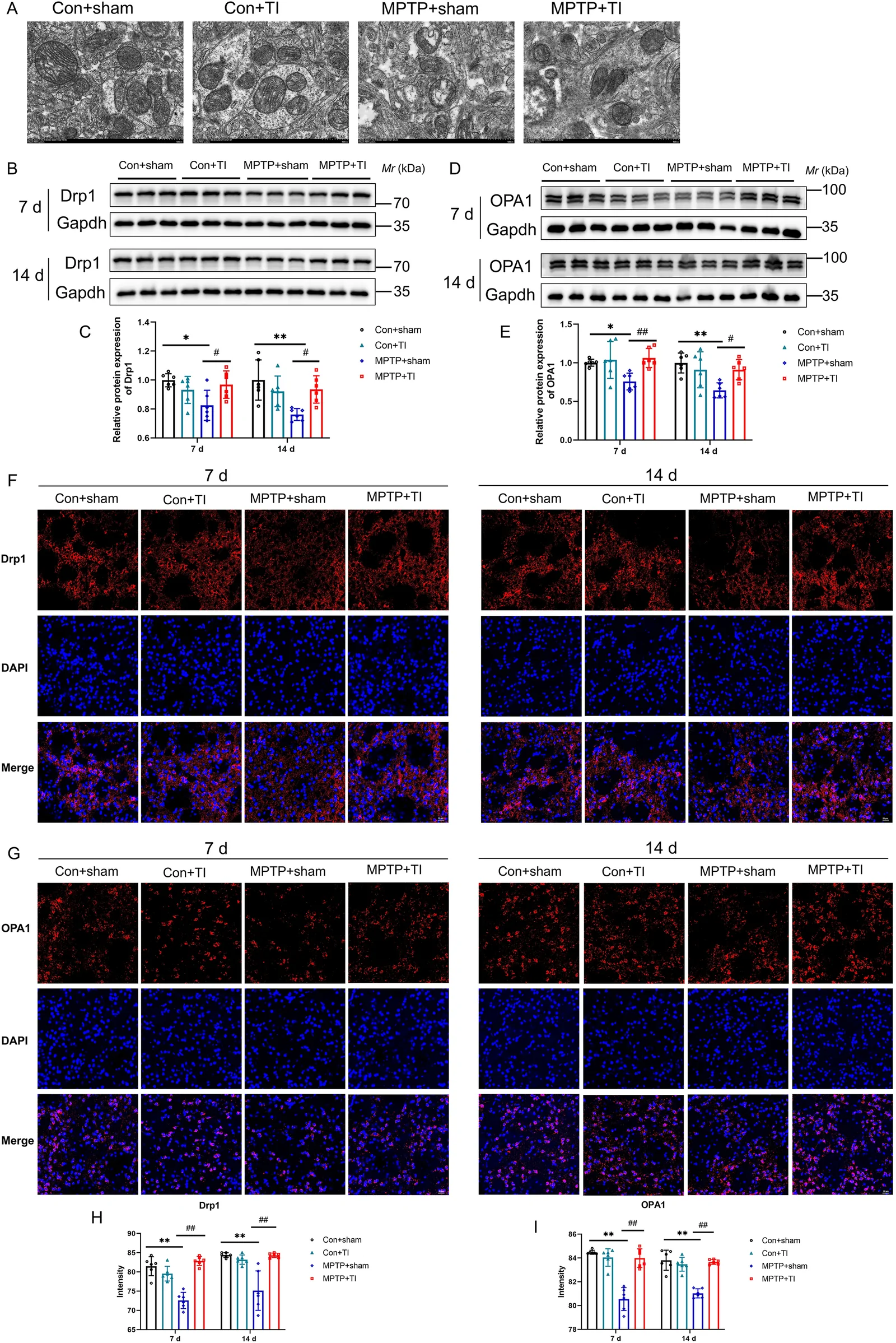
**Striatal TI stimulation normalizes mitochondrial morphology and dynamics in MPTP-treated mice.** (A) TEM image showing mitochondrial morphology in the striatum. Scale bar, 500 nm. N = 3. (B–E) WB and quantification of striatal Drp1 and OPA1 expressions. N = 6. (F–I) Confocal images and quantification of Drp1 and OPA1 in striatal coronal sections. Scale bar, 20 μm. N = 6. All data are presented as mean ± SD. ∗P < 0.05, ∗∗P < 0.01, vs Con + sham group; #p < 0.05, ##p < 0.01, vs MPTP + sham group.

Given the central role of mitochondrial dysfunction in dopaminergic neurodegeneration in PD [15,16], we next asked whether the neuroprotective effects of TI stimulation were associated with changes in mitochondrial morphology and dynamics. First, we used TEM to assess mitochondrial morphology in the striatum. In the MPTP + sham group, mitochondria appeared swollen and vacuolated, with a sparse matrix and disrupted cristae structure (Fig. 5A). By contrast, following 14 days of TI stimulation, mitochondrial ultrastructure was markedly improved, as indicated by a denser matrix and more arranged cristae structure, closely resembling that observed in the Con + sham group (Fig. 5A).

To further assess mitochondrial dynamics, we measured the expression of the key dynamin family proteins, Drp1 and OPA1, which regulate mitochondrial fission and fusion, respectively [37]. WB analysis showed that Drp1 and OPA1 protein levels in the striatum were reduced in MPTP + sham mice, whereas both 7-day and 14-day TI stimulation elevated their expression (Fig. 5B–E). IF staining of Drp1 and OPA1 showed similar changes in the striatum (Fig. 5F–I). Together, these findings indicate that striatal TI stimulation alleviates MPTP-induced abnormalities in mitochondrial morphology and dynamics.

### Striatal TI stimulation enhances mitophagy-related process, improves mitochondrial function and suppresses apoptosis in MPTP-treated mice

**Fig. 6:**
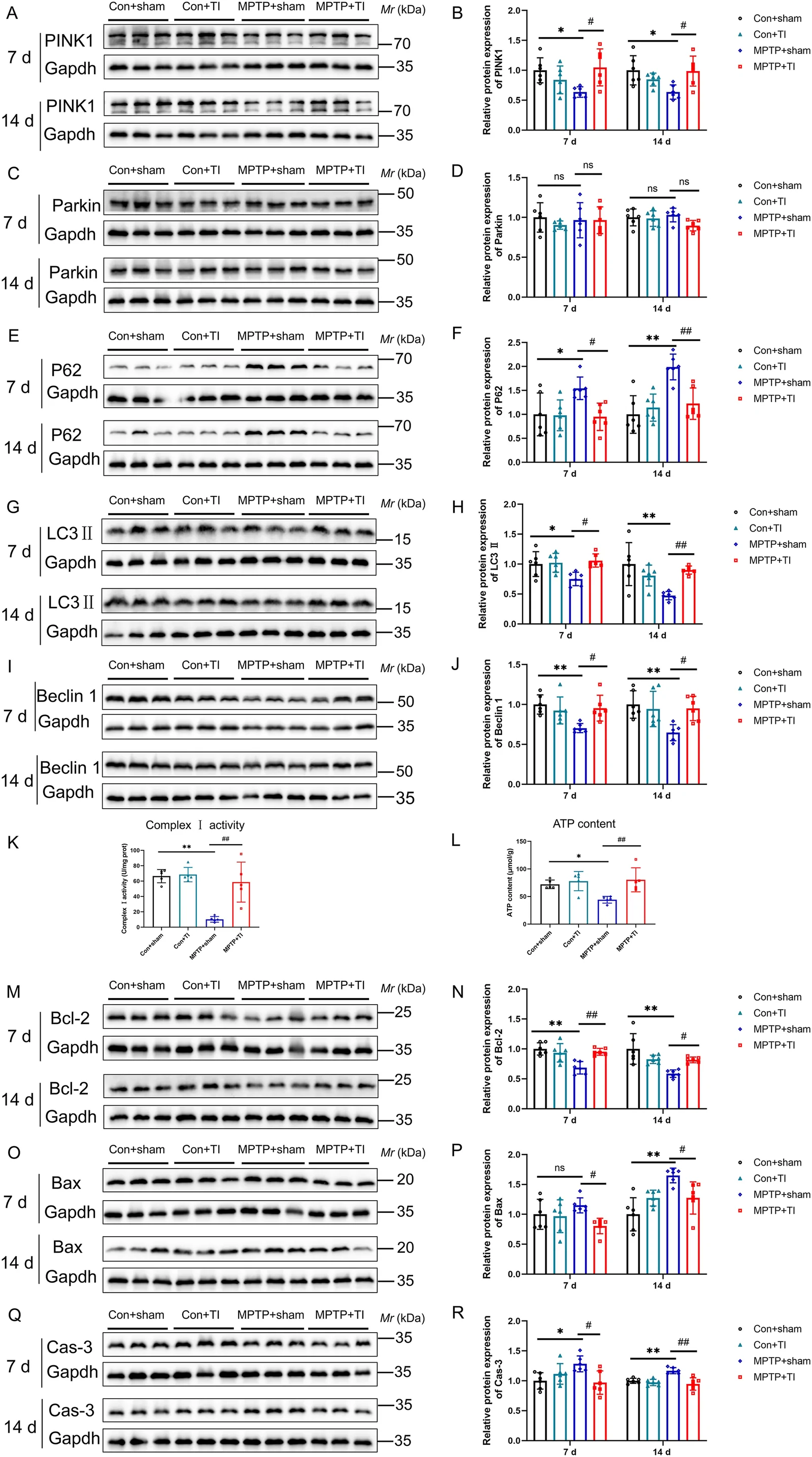
**Striatal TI stimulation enhances mitophagy-related process, improves mitochondrial function and suppresses apoptosis in MPTP-treated mice.** (A–J) WB and quantification of striatal mitophagy-related proteins: PINK1, Parkin, P62, LC3 II, and Beclin 1. N = 6. (K, L) Striatal complex I activity and ATP content. N = 5. (M–R) WB and quantification of striatal apoptosis-related proteins: Bcl-2, Bax, and Cas-3. N = 6. All data are presented as mean ± SD. ∗P < 0.05, ∗∗P < 0.01, vs Con + sham group; #p < 0.05, ##p < 0.01, vs MPTP + sham group.

To determine whether TI stimulation affects mitochondrial quality control, we examined proteins involved in mitophagy in the striatum. MPTP treatment reduced the levels of PINK1, LC3 II and Beclin 1, whereas both 7-day and 14-day TI stimulation increased their expression toward control levels (Fig. 6A, B, G-J). In parallel, TI stimulation decreased P62 levels (Fig. 6E and F). Parkin expression did not differ significantly among groups (Fig. 6C and D). These findings suggest that TI stimulation enhances mitophagy-associated processes in the striatum of MPTP-treated mice.

We next assessed mitochondrial function by measuring complex I activity and ATP content [38]. MPTP-treated mice showed marked reductions in both indices, indicating impaired mitochondrial respiratory function (Fig. 6K and L). 14-day TI stimulation significantly increased complex I activity and ATP levels compared with MPTP-treated mice, indicating improvement in mitochondrial bioenergetics.

Because impaired mitophagy and mitochondrial respiratory function can trigger mitochondria-dependent apoptosis [39], we further examined apoptosis-related proteins. Both 7-day and 14-day TI stimulation increased Bcl-2 expression and reduced Bax and Cas-3 levels in the striatum of MPTP-treated mice (Fig. 6M-R). Together, these results indicate that striatal TI stimulation enhances mitophagy-related processes, improves mitochondrial function, and reduces mitochondria-dependent apoptosis-related signaling in MPTP-treated mice.

### Mdivi-1 attenuated the behavioral, neuropathological, and mitochondrial effects of TI stimulation

**Fig. 7:**
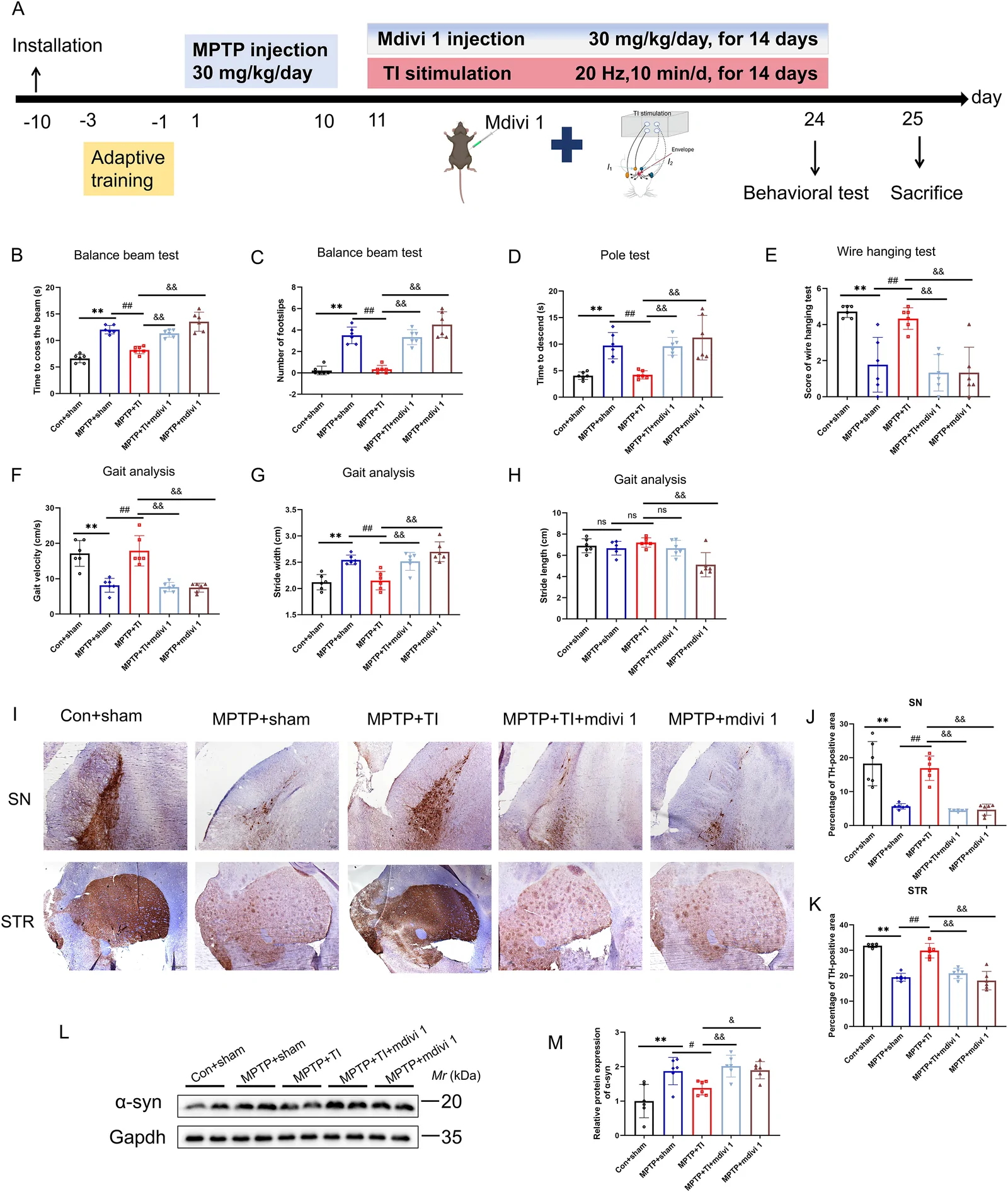
**Mdivi-1 attenuated TI stimulation-induced improvements in motor deficits and PD pathology.** (A) Schematic of experimental timeline. (B–H) Behavioral tests performed on day 14 of TI stimulation. N = 6. (I–K) IHC and quantification of TH-positive neurons in the SN and striatum. N = 6. (L, M) WB and quantification of striatal α-syn expression. N = 6. All data are presented as mean ± SD. ∗P < 0.05, ∗∗P < 0.01, vs Con + sham group; #p < 0.05, ##p < 0.01, vs MPTP + sham group; & p < 0.05, && p < 0.01, vs MPTP + TI group.

To further examine the mechanism underlying TI-mediated neuroprotection, Mdivi-1 was administered throughout the TI stimulation period to determine whether disruption of mitochondrial quality-control-related processes attenuated the effects of TI stimulation [[28], [29], [30]] (Fig. 7A). Mdivi-1 reduced the TI stimulation-induced improvements in motor performance in MPTP-treated mice, as assessed by the behavioral tests shown in Fig. 7B–H. In addition, the TI stimulation-induced preservation of TH-positive neurons and the reduction in α-syn expression were both significantly attenuated by Mdivi-1 treatment (Fig. 7I-M). These findings indicate that mitochondrial quality-control-related processes are functionally involved in the beneficial effects of TI stimulation on motor deficits and PD-like pathology.

**Fig. 8:**
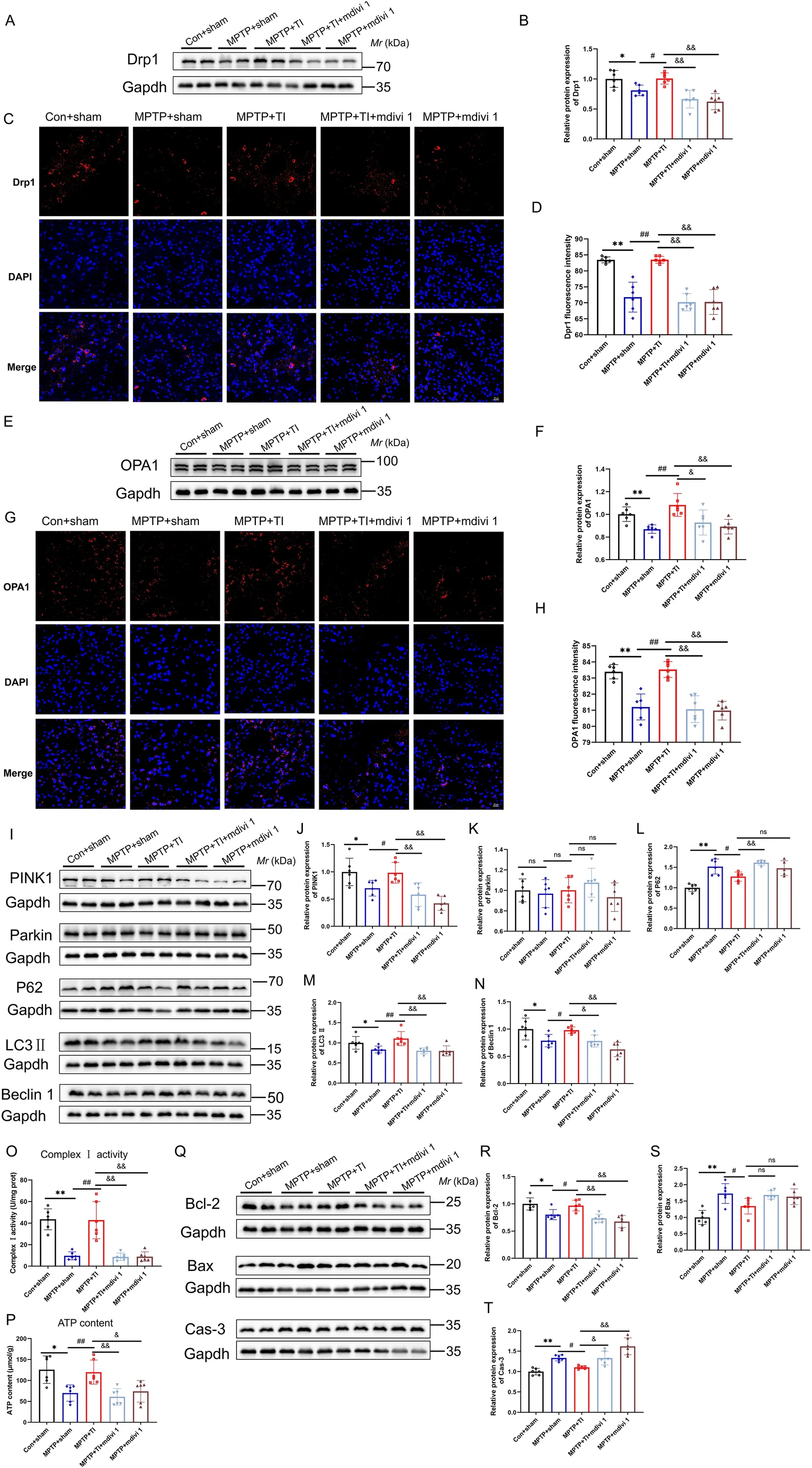
**Mdivi-1 attenuated TI stimulation-induced improvements in mitochondrial dysfunction.** (A, B, E, F) WB and quantification of striatal Drp1 and OPA1 expressions. N = 6. (C, D, G, H) Confocal images and quantification of Drp1 and OPA1 in striatal coronal sections. Scale bar, 20 μm. N = 6. (I–N) WB and quantification of striatal mitophagy-related proteins: PINK1, Parkin, P62, LC3 II, and Beclin 1. N = 6. (O, P) Striatal complex I activity and ATP content. N = 6. (Q–T) WB and quantification of striatal apoptosis-related proteins: Bcl-2, Bax, and Cas-3. N = 6. All data are presented as mean ± SD. ∗P < 0.05, ∗∗P < 0.01, vs Con + sham group; #p < 0.05, ##p < 0.01, vs MPTP + sham group; & p < 0.05, && p < 0.01, vs MPTP + TI group.

We next examined whether Mdivi-1 affected TI stimulation-associated changes in mitochondrial indices. Mdivi-1 markedly inhibited the TI stimulation-induced increases in striatal Drp1 and OPA1 expression in MPTP-treated mice (Fig. 8A–H). Similarly, TI stimulation-associated changes in PINK1, Parkin, P62, LC3 II, and Beclin 1 expression were attenuated by Mdivi-1 (Fig. 8I-N). Mdivi-1 also reduced the TI stimulation-induced recovery of striatal complex I activity and ATP content (Fig. 8O and P). In addition, Mdivi-1 blunted the TI stimulation-induced increase in Bcl-2 and the decreases in Cas-3 and Bax (Fig. 8Q-T). Collectively, these results suggest that mitochondrial quality-control-related processes contribute to the beneficial effects of TI stimulation in MPTP-treated mice.

### TI stimulation increases c-Fos expression in the striatal target region without evident tissue injury

**Fig. 9:**
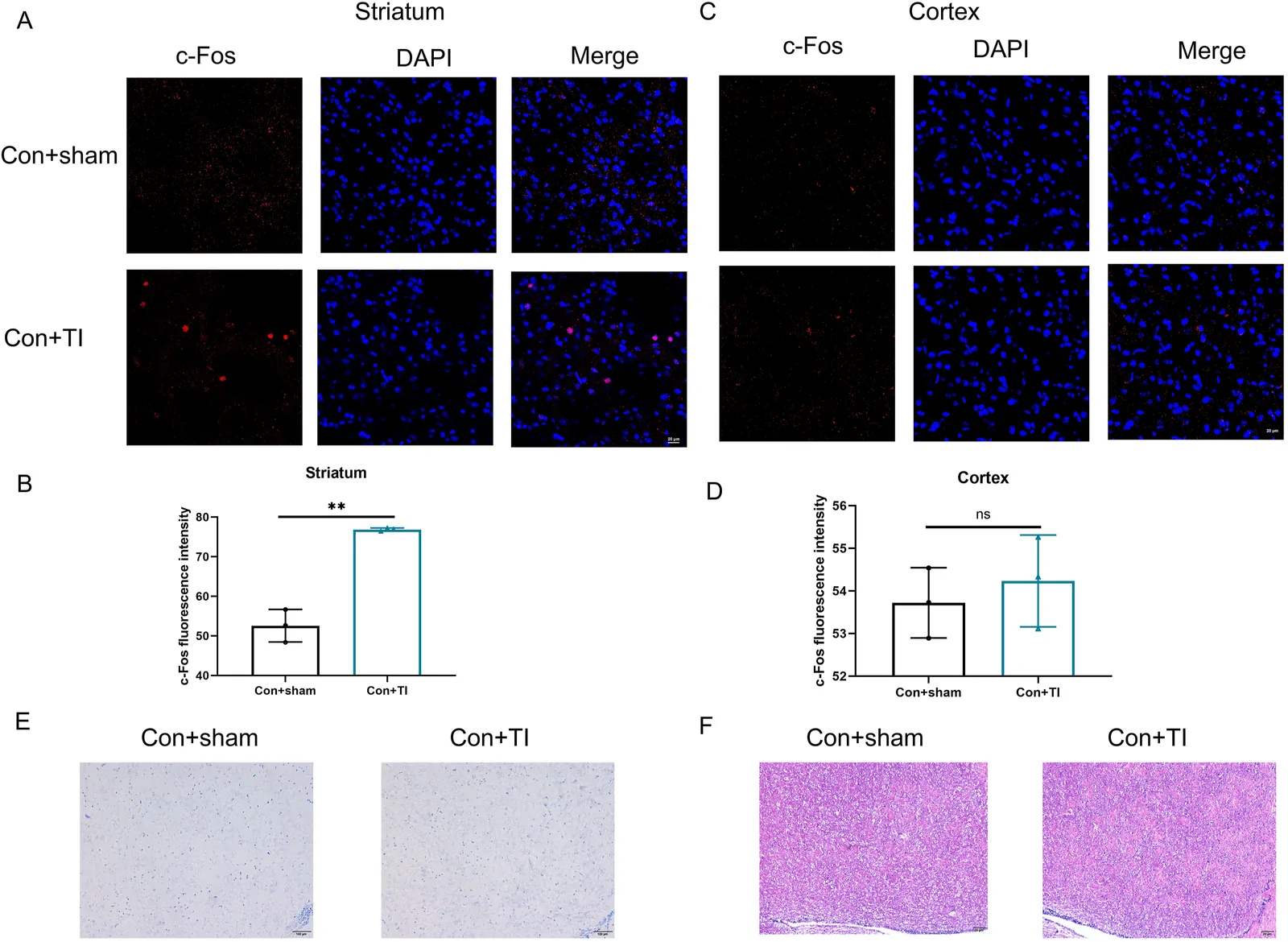
**TI stimulation increases c-Fos expression in the striatal target region without evident tissue injury.** Confocal images and quantification of c-Fos in ipsilateral striatal (A, B) and cortical (C, D) coronal sections. Scale bar, 20 μm. N = 3. Nissl (E) and HE staining (F) of striatum after 14 days of TI or sham stimulation. N = 3. All data are presented as mean ± SD. ∗P < 0.05, ∗∗P < 0.01, vs Con + sham group.

To assess the feasibility of TI stimulation, we examined neuronal activity in the striatum by IF staining for the neuronal activity marker c-Fos [40]. A single session of TI stimulation significantly increased c-Fos fluorescence intensity in the ipsilateral striatum (Fig. 9A and B), whereas no significant change was observed in the ipsilateral cortex (Fig. 9C and D). We next evaluated the safety of repeated stimulation. After 14 days of TI stimulation, Nissl staining showed comparable neuronal density in the striatum between groups (Fig. 9E), and HE staining revealed no hemorrhaging or tissue damage (Fig. 9F). These results support the feasibility and safety of the TI stimulation protocol used in this study.

## Discussion

**Fig. 10:**
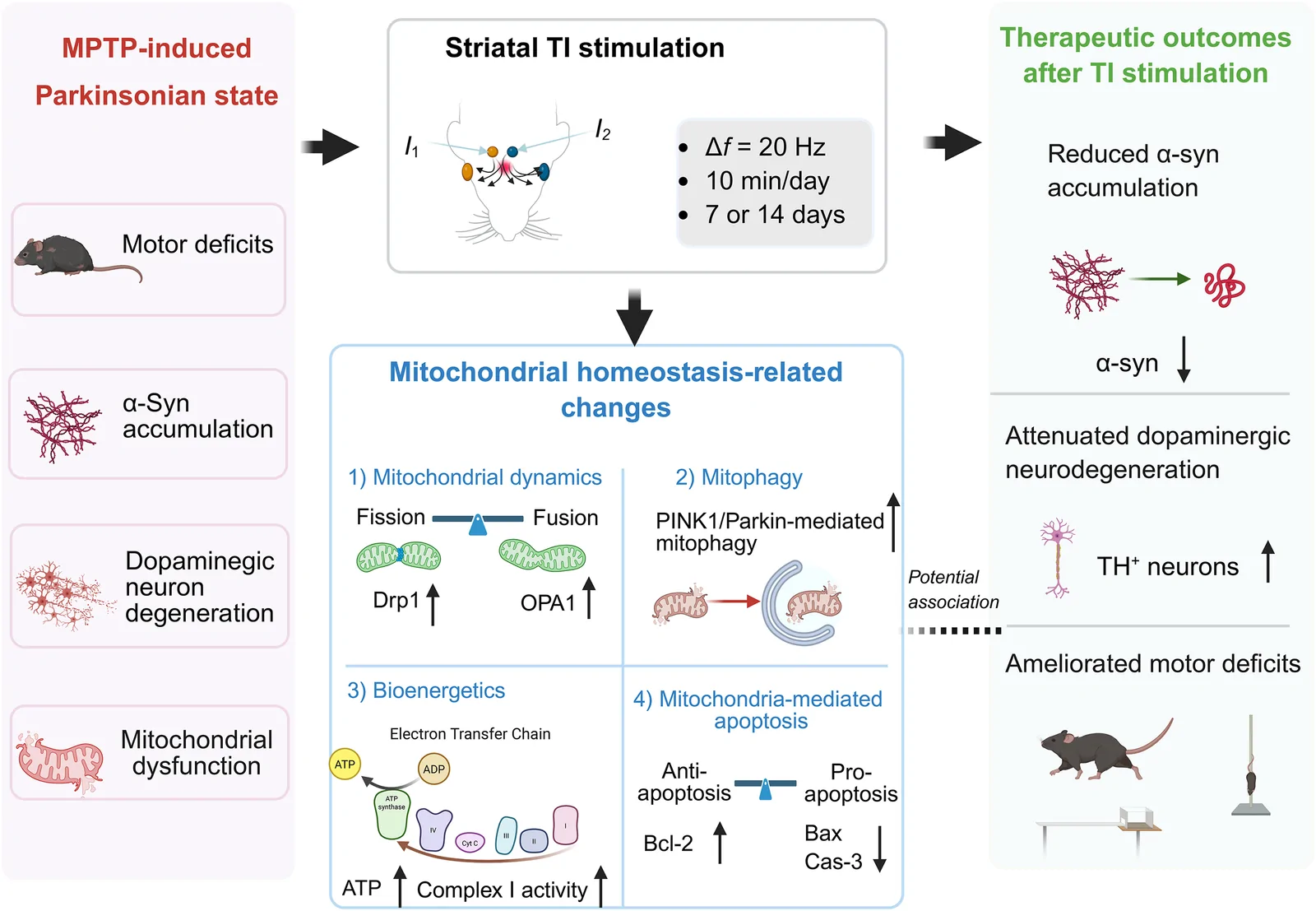
**Proposed model linking striatal TI stimulation with neuroprotective outcomes and mitochondrial homeostasis-related changes in MPTP-treated mice.** Striatal TI stimulation was associated with improved motor performance, attenuated dopaminergic neuron degeneration, reduced α-synuclein accumulation, and improvements in mitochondrial homeostasis-related processes in MPTP-treated mice. The relationships shown here represent a proposed working model and should not be interpreted as establishing a definitive causal sequence.

The present study provides evidence that striatal TI stimulation exerts both therapeutic and neuroprotective effects in an MPTP-induced mouse model of PD (Fig. 10). Specifically, TI stimulation improved motor deficits, attenuated dopaminergic neuron degeneration, and reduced α-syn accumulation. These benefits were accompanied by improvements in mitochondrial homeostasis-related processes, including normalization of mitochondrial morphology and dynamics, enhancement of mitophagy-related process, elevation of complex I activity, and suppression of mitochondria-dependent apoptosis. Importantly, Mdivi-1 substantially attenuated TI-associated effects, suggesting that mitochondrial quality-control processes may contribute to TI-mediated neuroprotection.

The striatum is a key input nucleus of the basal ganglia and plays a central role in the regulation of movement through integration of cortical, thalamic, and dopaminergic inputs [41]. Degeneration of nigrostriatal dopaminergic projections in PD disrupts striatal signaling and leads to abnormalities in basal ganglia-thalamo-cortical motor circuits [42]. In patients with PD, greater motor impairment has been associated with weaker connectivity between the anterior putamen and the midbrain, including the SN, while a more rapid decline in this connectivity predicts greater motor deterioration [43]. These provide a strong rationale for targeting the striatum with TI stimulation in PD treatment. In this study, we found that striatal TI stimulation improved motor performance in MPTP-treated mice, which aligns with holographic ultrasound stimulation results showing bilateral striatum stimulation reduced MPTP-induced motor deficits [40]. Collectively, these results support the striatum as a biologically relevant target for noninvasive neuromodulation in PD.

The TI stimulation parameters used in this study were selected based on prior human TI literature [9,[20], [21], [22], [23]] and our previous mouse studies [10,11,24]. Specifically, human studies targeting the M1 or striatum have commonly used carrier frequencies around 2 kHz, beta-range envelope frequencies, and stimulation durations of 10–30 min, and have reported modulation of motor performance [9,[20], [21], [22], [23]]. In our previous mouse studies, 7 consecutive days of TI stimulation using comparable parameters (carrier frequencies around 2 kHz, envelope frequencies of 10 or 20 Hz, 150 μA current, 30 s ramp-up/down, 10 or 20 min per day) enhanced motor performance and neurophysiological plasticity in the M1 and striatum [10,11,24]. We therefore selected 2 and 2.02 kHz carrier frequencies, a 20 Hz offset, 150 μA current, and 10 min/day stimulation in the present study to test whether a previously effective TI framework could also exert therapeutic and neuroprotective effects in the MPTP model. The 7-day and 14-day treatment schedules were included to evaluate both shorter-term and more sustained effects.

The rationale for selecting Δ*f* = 20 Hz in the present study differs from that of the 130 Hz TI protocol previously investigated in PD, which was informed by the high-frequency stimulation commonly used in DBS [12,44]. Our choice of 20 Hz was based on previous motor-related TI studies and the relevance of beta-range activity to motor control and cortico-basal ganglia interactions [45]. These distinct rationales do not indicate whether 20 and 130 Hz TI engage shared or distinct mechanisms. Because envelope frequencies were not directly compared, our findings support the efficacy of the complete 20 Hz protocol but do not establish its optimality or frequency specificity. It therefore remains unknown whether stimulation at 10, 80, or 130 Hz would produce comparable behavioral, neuropathological, or mitochondrial effects.

Abnormal beta-band activity within the basal ganglia-thalamocortical network is associated with bradykinesia and the antikinetic state of PD [42,46], and has also been reported in MPTP-induced parkinsonian models [47,48]. Because the envelope frequency used here lies within the beta range, modulation of pathological network dynamics represents a plausible upstream contributor to the observed effects. Previous studies provide indirect support for this possibility. Beta-range noninvasive stimulation has been shown to modulate oscillatory and motor readouts in PD-related contexts [49]. STN-targeted TI stimulation has also been reported to reduce beta oscillatory activity in patients with PD, although that study used a different stimulation target and envelope frequency (Δ*f* = 130 Hz) from those used here [44]. In addition, a primate TI study that included a 20 Hz envelope frequency demonstrated that TI can alter spike timing and disrupt ongoing oscillatory synchrony [50]. However, we did not record local field potentials or other oscillatory measures and therefore cannot determine whether pathological beta-like activity was present or modified by striatal TI stimulation. Network-level modulation should consequently be regarded as a plausible but untested upstream mechanism, whereas the present data more directly support an effect on mitochondrial homeostasis.

An important finding of this study is that 14 days of TI stimulation produced greater behavioral improvement and preservation of TH-positive neurons than 7 days of stimulation, suggesting that repeated exposure may generate progressively stronger or more persistent functional benefits. A similar duration-dependent pattern has been reported for low-intensity focused ultrasound in MPTP-treated mice, with 14-day stimulation producing greater motor improvement than 7-day stimulation [32]. However, both TI treatment durations improved several mitochondrial indices, whereas the behavioral and dopaminergic neuronal outcomes were more pronounced after the longer intervention. This partial dissociation suggests that mitochondrial regulation may not fully account for the duration-dependent behavioral response. Prolonged TI stimulation may additionally engage processes such as neural plasticity [51] or modulation of neuroinflammation [52].

Mitochondrial dynamics are essential for maintaining mitochondrial integrity and cellular homeostasis [53], and disruption of the balance between fission and fusion has been implicated in PD pathogenesis [54]. In the present study, MPTP exposure was associated with abnormal mitochondrial morphology and reduced expression of proteins involved in mitochondrial dynamics, whereas TI stimulation partially corrected these abnormalities. Because proper mitochondrial dynamics facilitate segregation of damaged mitochondrial components and support subsequent quality-control processes, the normalization of mitochondrial morphology and dynamics may represent an early and important step in the neuroprotective effects of TI stimulation.

In addition to improving mitochondrial morphology, TI stimulation enhanced mitochondrial quality control and function in MPTP-treated mice. We observed activation of PINK1/Parkin-related mitophagy, increased complex I activity, and reduced mitochondria-dependent apoptotic signaling following TI stimulation. These changes are important because defective mitophagy leads to accumulation of dysfunctional mitochondria, energy failure, and oxidative stress, all of which contribute to dopaminergic neuron vulnerability in PD [55,56]. By facilitating removal of damaged mitochondria and improving respiratory chain function, TI stimulation may reduce mitochondrial stress and thereby limit apoptotic cell death. This mechanistic interpretation is consistent with the observed preservation of dopaminergic neurons in TI-stimulated mice.

Another notable finding is that striatal TI stimulation reduced α-syn accumulation in MPTP-induced mice. Given the well-established reciprocal relationship between α-syn aggregation and mitochondrial dysfunction, this result suggests that TI stimulation may interrupt a pathogenic cycle in which mitochondrial damage and α-syn pathology mutually exacerbate one another [57,58]. Importantly, Mdivi-1 largely reversed the beneficial effects of TI stimulation on motor performance, neuropathological changes, and mitochondrial indices, indicating that mitochondrial quality-control-related pathways are functionally involved in the actions of TI stimulation. However, because Mdivi-1 may affect multiple mitochondrial processes, these results should be interpreted as strong mechanistic support rather than definitive proof of a single pathway.

Our findings identify mitochondrial quality control as an important downstream contributor to TI-mediated neuroprotection but do not establish mitochondria as the primary direct biophysical target of TI stimulation. Instead, in light of current TI literature [7,50,[59], [60], [61]], we favor a model in which TI primarily modulates pathological striatal neuronal and/or circuit activity, which may then secondarily influence activity-dependent calcium signaling, metabolic demand, and stress-response pathways, thereby promoting mitochondrial homeostasis. This interpretation is consistent with recent evidence suggesting that TI effects may arise from nonlinear membrane rectification and excitatory-inhibitory network interactions [59,60]. Moreover, TI appears to preferentially perturb spike timing and phase locking rather than strongly drive firing rates [50]. Consistent with this interpretation, in our study, TI did not significantly alter the tested mitochondrial markers under physiological conditions, but instead corrected MPTP-induced mitochondrial abnormalities. Together with the Mdivi-1 findings, these results support mitochondrial homeostasis as a potential downstream component of TI-mediated neuroprotection in the diseased state rather than an established direct target of stimulation. Notably, the present study does not determine whether action potential generation is required for the mitochondrial effects of TI stimulation; subthreshold modulation remains a biologically plausible alternative or complementary mechanism. We also cannot exclude additional contributions from intracellular calcium dynamics, metabolic regulation, stress-response pathways, or non-neuronal cellular effects [59,[62], [63], [64]]. These upstream mechanisms should be dissected in future studies using electrophysiological recording, calcium imaging, and cell type-specific approaches.

Several limitations should be acknowledged. First, we did not directly measure or computationally model the electric field generated by our TI montage. Thus, the carrier-field magnitude and modulation-envelope amplitude in the striatum and surrounding regions remain unquantified in the present preparation, and the present data cannot determine whether the stimulation was subthreshold or suprathreshold. Accordingly, selective delivery of the electric field to the striatum cannot be established from the present data. Comparable stimulation paradigms in our previous mouse studies produced measurable neurophysiological effects, including increased field excitatory postsynaptic potentials (fEPSP) and long-term potentiation (LTP) in M1, enhanced calcium signals during motor behavior test, and increased striatal fEPSP [11,24]. Together with the present c-Fos results, these findings provide indirect evidence of biological engagement but cannot substitute for direct field quantification or demonstrate that neurons were driven to fire at Δ*f*. Future studies should combine computational modeling, electrophysiology, and direct field measurements to address these issues more rigorously.

Second, the experimental design did not directly distinguish the contributions of the kilohertz-frequency carriers and the 20 Hz interference envelope. Previous findings nevertheless support the functional relevance of the interference structure. Grossman et al. reported no detectable neuronal activation with either a single 2 kHz stimulus or two equal-frequency 2 kHz currents (Δ*f* = 0) under their acute experimental conditions, whereas temporally interfering fields induced neural and behavioral responses [7]. Moreover, our previous study found different motor and neuroplastic effects with Δ*f* = 10 and 20 Hz under otherwise comparable stimulation conditions, indicating that biological responses can vary with the frequency difference [11,24]. However, these observations do not determine the relative contributions of the carrier currents and the envelope in the present MPTP model. The absence of an acute response to a particular carrier-only condition does not exclude carrier-related effects during repeated stimulation, and comparison of two Δ*f* values does not provide a no-envelope baseline. Therefore, the present findings establish the efficacy of the complete TI protocol and support a functional contribution of its low-frequency temporal structure, but do not demonstrate that the effects were mediated primarily or exclusively by the 20 Hz envelope. The Mdivi-1 experiments identify mitochondrial quality-control processes as a downstream mechanism but do not resolve which waveform component initiated the response. Future studies using field-matched no-envelope controls and systematic Δ*f* comparisons are required to distinguish these contributions.

Third, the study was conducted in a subacute neurotoxin-induced mouse model, which does not fully recapitulate the progressive and heterogeneous nature of human PD [65]. The durability of the observed effects after stimulation cessation also remains to be established. In addition, behavioral assessments were conducted by two investigators who were aware of the group assignments, which may have introduced observer bias despite the use of the same trained investigators, standardized testing procedures, and consistent testing times. Finally, several potential confounding factors should also be considered. Although the sham-stimulated mice underwent the same anesthesia, handling, electrode placement, and experimental schedule as the TI-stimulated mice, repeated anesthesia and procedure-related stress cannot be completely excluded as contributors to the observed effects. Moreover, the improvement in motor activity may have secondarily affected mitochondrial function, autophagy-related processes, and apoptotic signaling; therefore, these molecular changes should not be interpreted exclusively as direct consequences of striatal TI stimulation. Although the sham procedure controlled for procedural factors, it may not fully account for peripheral or other nonspecific effects of active electrical stimulation or current spread beyond the intended target region. Future studies using awake stimulation, active or off-target stimulation controls, and direct assessment of stress-related parameters are needed to further clarify the target specificity and underlying mechanisms of TI stimulation.

In conclusion, striatal TI stimulation ameliorated motor impairment and attenuated dopaminergic neurodegeneration in an experimental model of PD, with the observed benefits being associated with improvements in mitochondrial homeostasis-related processes. The findings extend the potential biological effects of TI stimulation beyond acute symptomatic neuromodulation and implicate mitochondrial quality-control processes as a potential contributor to the observed neuroprotective effects. Nevertheless, the upstream neural mechanisms, electric-field characteristics, waveform-component specificity, and optimal stimulation frequency remain to be determined before the therapeutic potential of this approach can be fully defined.

## Data availability

Data used in this manuscript are available under reasonable request.

## CRediT authorship contribution statement

**Jin Peng:** Writing – review & editing, Writing – original draft, Methodology, Data curation. **Ziyu Wang:** Investigation, Data curation. **Yu Liu and Xiaohui Wang:** Writing – review & editing, Supervision, Funding acquisition.

## Funding

This work was supported by the 10.13039/501100001809National Natural Science Foundation of China (Grant numbers 11932013).

## Declaration of competing interest

The authors declare that they have no known competing financial interests or personal relationships that could have appeared to influence the work reported in this paper.
